# Impact of STAT/SOCS mRNA Expression Levels after Major Injury

**DOI:** 10.1155/2014/749175

**Published:** 2014-02-06

**Authors:** M. Brumann, M. Matz, T. Kusmenkov, J. Stegmaier, P. Biberthaler, K.-G. Kanz, W. Mutschler, V. Bogner

**Affiliations:** Department of Trauma Surgery, Ludwig Maximilians University Hospital Munich, Nußbaumstraße 20, 80336 Munich, Germany

## Abstract

*Background*. Fulminant changes in cytokine receptor signalling might provoke severe pathological alterations after multiple trauma. The aim of this study was to evaluate the posttraumatic imbalance of the innate immune system with a special focus on the *STAT/SOCS* family. *Methods*. 20 polytraumatized patients were included. Blood samples were drawn 0 h–72 h after trauma; mRNA expression profiles of IL-10, STAT 3, SOCS 1, and SOCS 3 were quantified by qPCR. *Results*. IL-10 mRNA expression increased significantly in the early posttraumatic period. STAT 3 mRNA expressions showed a significant maximum at 6 h after trauma. SOCS 1 levels significantly decreased 6 h–72 h after trauma. SOCS 3 levels were significantly higher in nonsurvivors 6 h after trauma. *Conclusion*. We present a serial, sequential investigation in human neutrophil granulocytes of major trauma patients evaluating mRNA expression profiles of IL-10, STAT 3, SOCS 1, and SOCS 3. Posttraumatically, immune disorder was accompanied by a significant increase of IL-10 and STAT 3 mRNA expression, whereas SOCS 1 mRNA levels decreased after injury. We could demonstrate that death after trauma was associated with higher SOCS 3 mRNA levels already at 6 h after trauma. To support our results, further investigations have to evaluate protein levels of STAT/SOCS family in terms of posttraumatic immune imbalance.

## 1. Introduction

The immunological response to severe multiple trauma remains a critical challenge to human health. Traumatic injury has been known to induce substantial alterations in immune function, which compounds both proinflammatory activation known as systemic inflammation response syndrome (SIRS) and anti-inflammatory reaction leading to immunosuppression termed as compensatory anti-inflammatory response syndrome (CARS). In multiple trauma, both syndromes occur simultaneously, which was recently described as mixed antagonist response syndrome (MARS) [1]. This serious imbalance of cell-regulated innate immune system often contributes to posttraumatic multiple organ dysfunctions or even multiple organ failure. With a cell fraction of 50–60% of all leukocytes, polymorphonuclear leukocytes have been shown to be key effector cells within the innate inflammatory immune reaction [2]. Characteristically, severe trauma leads to immediate hyperinflammatory response with neutrophil activation followed by continuous proinflammatory interleukin secretion (e.g., IL-6), which usually induces an upregulation of further major anti-inflammatory mediators, such as IL-10 [3]. Timing, control, and extent of this mediator-associated acute-phase response to injury appear to distinctly influence these patients' further clinical course. Previous mRNA expression studies of our group could already investigate specific mRNA expression patterns and activated signalling pathways in monocytes after major trauma. One of our precedent genome-wide microarray analyses could identify MAP (mitogen-activated protein) kinase p38, JNK (Janus kinase), and c-Jun to play an important role in trauma patients in the early posttraumatic period and could link the increase of gene transcription in these factors to unfavorable outcome [4, 5]. In this context, the protein family of Signal Transducers and Activators of Transcription (STAT) and Suppressors of Cytokine Signalling (SOCS) came into the focus of interest. Through phosphorylation on conserved serine and tyrosine residues STATs are activated by the Janus kinase and MAP kinase [6]. After their activation the STAT proteins function as sensible sensors responding to cellular stress and modulators of cytokine signalling by regulating their gene expression via translocation to the nucleus [7]. STAT proteins all contain a transcriptional transactivation domain as well as a phosphorylation domain for JAKs and MAPKs, which are both substantial for maximal STAT function [6]. Tyrosine phosphorylation of STAT proteins occurs in response to cytokine receptors through Janus kinases (JAKs), which is commonly known as the JAK-STAT pathway [8]. Following these current opinions, it is essential to gain more insight into the complex regulation of underlying pathways in case of posttraumatic cytokine disorder, as most cytokines have been shown to use the Janus kinase signal transducers and activators of transcription pathway [9]. Obviously, STAT 3 is induced by IL-6, closely interacts with IL-10, and is associated with the acute-phase reaction in the human liver as well as with the maintenance of sepsis [10, 11]. SOCS genes are one of their most considerable targets; they suppress further cytokine receptor signalling generally in a negative feedback loop [9, 12]. SOCS proteins also bind to JAK; particularly SOCS 1 and SOCS 3 have been evaluated to be strong inhibitors of JAKs.

The aim of this study was to evaluate the posttraumatic disorder of the innate immune system with a special focus on the *STAT/SOCS* protein family. We examined mRNA expression profiles of IL-10, STAT 3, SOCS 1, and SOCS 3 in neutrophil granulocytes after blunt multiple trauma in the early posttraumatic period and correlated their levels to distinct clinical conditions. Therefore, patients were distributed to different subgroups in regard to injury severity and outcome.

## 2. Material and Methods

### 2.1. Study Design

Patients after blunt multiple trauma (NISS > 16) aged between 18 and 70 years who were admitted to our level 1 trauma centre within 90 minutes after trauma were included in this study. Signed informed consent was obtained from each patient or his/her legal representative over the course of time; Ethical Committee Permission was obtained from Ludwig Maximilians University, Munich, Germany (reference number: 012/00). Patients who did not survive the first 24 hours after trauma and patients with an isolated brain injury were excluded. Blood samples were drawn immediately after admission and again 6, 12, 24, 48, and 72 hours after trauma. Patients were assigned to different groups regarding the two following clinical conditions: injury severity assessed by New Injury Severity Score (with our patient collective's median NISS of 41 points as cutoff) and outcome (survival of 90 days after trauma) [13]. Various clinical data such as age, gender, injury severity, pattern of injury, and parameters of cardiovascular function were recorded.

### 2.2. Sample Collection

A total volume of 30 mL of EDTA whole blood was drawn. RBC lysis was performed by adding 42.5 mL of blood cell lysis buffer (155 mM NH_4_Cl, 10 mM KHCO_3_, and 0.1 mM EDTA, pH 7.2) to each 7.5 mL of EDTA-anticoagulated whole blood. Subsequently samples were centrifuged for 10 min at 450 g. To achieve quantitative RBC removal from the cell pellets, two additional lyses and centrifugation steps were performed. The resulting cell pellets were then resuspended in 500 mL of phosphate-buffered saline PBS/EDTA and were further diluted for granulocyte separation as described below.

### 2.3. Granulocyte Separation and RNA Isolation

Granulocyte cell separation was performed using a magnetic-activated cell sorting (MACS) method, which is a special positive immune magnetic technique for separation of various cells depending on their surface antigens. We used anti-CD15 antibody-coated micromagnetic beads for isolation of neutrophil granulocytes, as neutrophil granulocytes express CD15 antigen on their surface. Neutrophil granulocytes attach to CD15 antibody magnetic beads and can thereby be separated positively. In detail, cells were diluted in 800 mL of PBS/EDTA buffer and incubated with 200 mL of anti-CD15 antibody-coated micromagnetic beads (Miltenyi Biotech, Auburn, CA) for 15 min at 4°C. After incubation, the volume was adjusted to 15 mL using PBS/EDTA buffer, and the cells were pelleted by centrifugation for 10 min at 450 g. The cell pellets were resuspended in 500 mL of PBS/EDTA buffer and were added to a preequalized high-gradient magnetic separation column using the magnetic cell separator Mini-MACS system (Miltenyi Biotech). The column was washed three times. The cell suspension was lysed in 2 mL of RLT buffer (Qiagen, Hilden, Germany) containing 0.1% (v/v) beta-mercaptoethanol and was stored at –80°C. Total RNA preparation from lysed granulocytes was performed using the RNeasy Midi Kit according to the instructions of the supplier (Qiagen, Hilden, Germany). Nucleic acids were digested by DNase according to manufacturer's guidelines *(RNase-free DNase Set; Qiagen, Hilden, Germany)*. Concentration of isolated RNA was determined by spectrophotometry (260 nm, Biophotometer, Eppendorf, Hamburg, Germany). As the integrity of total RNA has a distinct influence on qPCR accuracy, overall quality of RNA preparation was assessed by electrophoresis on a denaturing agarose gel.

### 2.4. Quantitative Real-Time PCR (qPCR)

An aliquot of 8.2 *μ*L standardized to 1 *μ*g of RNA was reverse transcribed with avian myeloblastosis virus-reverse transcriptase (AMV-RT) and oligo-p(dT)_15_-primer following manufacture's instruction (1st Strand cDNA Synthesis Kit, Roche, Mannheim, Germany). After denaturation (Thermocycler, 65°C for 15 min), samples were iced for 5 minutes and incubated with manufacture's master-mix (11.8 *μ*L including MgCl_2_, desoxynucleotid-mix, oligo-p(dT)_15_-primer, AMV reverse transcriptase and RNase inhibitor). The obtained cDNA was diluted 1/25 with water and 10 *μ*L was used for amplification. The quantitative analysis of target gene expression was performed on a LightCycler by real-time PCR using the Light Cycler Fast Start DNA Master SYBR Green I Kit according to the manufacture's instructions (Roche, Mannheim, Germany). Standard and primer were designed by Search LC (Heidelberg, Germany; LightCycler Primer Sets). qPCR was performed by denaturation and polymerase activation (95°C for 10 min), amplification of qPCR products in a 45-cycle one-step PCR including denaturation (95°C, 10 s)/annealing (68°C–58°C, −0,5°C/cycle)/extension (72°C, 16 s) for each circle. The results of negative control samples (NTC) were set as baseline level. Results of qPCR were standardized to this baseline level and given as copies/50 ngRNA. Specificity of the amplification products was verified by melting curve analysis combined with agarose gel electrophoresis. Regarding PCR efficiency standard curves were prepared for each gene. The slope of the standard curve marks qPCR efficiency; slope varied from −3.3 to −3.5 in our study.

### 2.5. Statistical Analysis

Data were statistically analysed using Friedman Repeated Measures Analysis of Variance on Ranks as a method to compare several dependant spot tests to test significant differences regarding the time points (0 h, 6 h, 12 h, 24 h, 48 h, and 72 h). In case of significant differences we used Student-Newman-Keuls test (SNK test). Subgroups were tested using the non-parametric Mann-Whitney-*U* test (*U* test). The results were declared as mean values (mean) with standard error of the mean (SEM) and were considered statistically significant when *P* < 0.05.

## 3. Results

### 3.1. Clinical Baseline Characteristics

20 polytraumatized patients (13 male 7 female) presenting with a NISS > 16 points were enrolled in this study according to the abovementioned inclusion criteria. The patients' age raged between 18 and 70 years (mean ± SEM: 40 ± 2.8 years) with a NISS of 20 to 75 points (mean ± SEM: 40.5 ± 3.2). Four patients (20%) died; 16 patients (80%) survived observed period of 90 days.

Retrospectively, we separated the patient collective into two groups regarding the injury severity; that is, we used a cutoff value of 41 points according to the median NISS in all patients (group 1: NISS < 41, *n* = 9; group 2: NISS ≥ 41, *n* = 11). There were 9 patients with a NISS less than 41 points; 7 of them were male; two female. Mean age was 45.5 ± 4.0 (mean ± SEM) years; mean NISS was 27.1 ± 2.1 (Mean ± SEM) points. Two patients in this group died within 90 days after trauma. There were 11 patients with a NISS equal to or more than 41 points; six of them were male and five female. Mean age was 37.3 ± 3.5 (MW ± SEM) years; mean NISS was 52.4 ± 2.4 (mean ± SEM) points. In a second step, we separated the collective into two groups (group A: survivors, *n* = 16; group B: nonsurvivors, *n* = 4) regarding the clinical parameter outcome (90-day survival after trauma). 16 patients survived (11 male and 5 female with a mean age of 38.3 ± 2.9 (mean ± SEM) years). The mean NISS was 40.3 ± 3.7 (mean ± SEM) points in this subcollective. Four patients deceased during this time frame (2 male and 2 female with a mean age of 48 ± 6.4 (mean ± SEM) years). Mean NISS in this subgroup was 41.3 ± 6.9 (mean ± SEM) points. Clinical data of all 20 patients are condensed in Table 1.

### 3.2. IL-10 mRNA Expression Levels (Figure 1)

Our study's results showed significantly higher mRNA expression levels of IL-10 in granulocytes in all patients during the course of the first three days after multiple trauma in contrast to the initial value examined on admission. IL-10 mRNA expression was significantly higher in patients presenting with a NISS less than 41 points (group 1) 24, 48, and 72 h after trauma. Both groups (groups 1 and 2) showed a significant raise of IL-10 mRNA expression levels at all examined time points compared to the values detected on admission (0 h). There were no significant differences between survivors (group A) and nonsurvivors (group B).

### 3.3. STAT 3 mRNA Expression Levels (Figure 2)

STAT 3 expression levels increased in the early posttraumatic period with a significant peak at 6 h after trauma. Considering the clinical parameters injury severity and outcome, STAT 3 mRNA levels were higher in patients with less injury severity (group 1) than in more severely injured patients (group 2). Patients, who died within the followup of 90 days after the traumatic event (group B), showed a lower STAT 3 expression directly (0 h) after trauma compared to the subcollective of survivors (group A). However, the expression levels showed just tendencies and no significant differences.

### 3.4. SOCS 1 mRNA Expression Levels (Figure 3)

Regarding the course of time (6–72 h), mRNA expression of SOCS 1 significantly decreased compared to the mRNA expression determined initially after trauma (0 h). There were no significant differences of SOCS 1 expression profiles after dividing the patients into different subgroups according to the clinical conditions injury severity and outcome.

### 3.5. SOCS 3 mRNA Expression Levels (Figure 4)

In the observed period of 72 h after polytrauma, mRNA expression of SOCS 3 was high on admission and followed by a decrease in all patients. Six hours after the traumatic event, SOCS 3 mRNA expression was significantly higher in patients who died (group B) within 90 days after trauma in comparison to those who survived (group A).

## 4. Discussion

Major trauma entails the development of severe systemic inflammation reaction and complex immune disorder [14]. In this context, fulminant changes in cytokine receptor signalling may provoke severe pathological alterations, particularly in regard to immune and blood cells. Following current opinion, various extracellular proteins, binding to cell-surface receptors, are able to induce such changes by altering their gene expression profile. Therefore, we evaluated the effect of multiple injuries on mRNA expression of IL-10, STAT 3, SOCS 1, and SOCS 3 in PMNs. Main findings in this presented study are a significant increase of IL-10 mRNA expression after blunt multiple trauma in all patients as well as a significant early induction of STAT 3 mRNA six hours after trauma. Furthermore, SOCS 1 mRNA expression levels significantly decreased over the time compared to SOCS 1 levels measured on admission. Regarding the subgroup of survivors, SOCS 1 mRNA levels also significantly decreased in the posttraumatic observation period (6 h–72 h) in comparison to SOCS 1 mRNA expression detected on admission. Considering SOCS 3 mRNA levels, our study could show a significant correlation to clinical outcome already at 6 h after trauma as nonsurvivors expressed significantly higher levels of SOCS 3 mRNA than survivors.

Both, STATs and SOCSs, have been shown to play a pivotal role in case of inflammation, trauma, and burn injury but have not yet been examined in case of multiple trauma [9].

### 4.1. IL-10 mRNA Expression

We can state a significant increase of IL-10 mRNA expression after blunt multiple trauma in all patients; this dynamic of posttraumatic IL-10 production is already described in polytraumatized patients [15]. Comparing group 1 and group 2, mRNA expression was significantly higher in patients suffering from moderate injury. This result is contrary to those in other examined trauma patient collectives. Expectedly, IL-10 mRNA expression profiles would have correlated with the magnitude of injury [15, 16]. But still, precise nature of IL-10 response following traumatic injury or shock remains controversial. Some studies suggest that high IL-10 levels are deleterious mediating immunosuppression after trauma, whereas others consider high IL-10 levels to be beneficial [17]. The potentially beneficial underlying mechanism includes the reduction of proinflammatory response in order to optimize tissue repair and healing potential of the affected organism—with the need of an executed balance between pro- and anti-inflammatory mediators. Possibly, higher levels of anti-inflammatory IL-10 mRNA expression levels in less injured patients (group 1) might reflect the organism's successful attempt to reach a level of temporary and well-balanced posttraumatic systemic inflammatory response in this subcollective. Thus, our study's results support the concept that early IL-10 overexpression might be important in damping the postinjury immune alteration.

### 4.2. STAT 3 mRNA Expression

STAT 3 mRNA expressions showed increasing levels in the early posttraumatic period with the significant highest level six hours after the traumatic event. This early induction of STAT 3 mRNA expression is in accordance with the known posttraumatic concentration increase of various cytokines, such as IL-6, in polytrauma patients [18]. Activated cytoplasmic STAT 3 rapidly accumulates in the nucleus in response to IL-6 stimulation [19, 20]. Correlating STAT 3 mRNA expression profiles with injury severity, our results do not show significant differences. But, by tendency, our results show higher mRNA levels in less severely injured patients (group 1). This result is in line with the aforementioned results of IL-10 mRNA expression levels. In both cases, mRNA expression levels are higher in patients presenting with a NISS less than 41 points; this result may be interpreted in the same way as before. Quite possibly, the STAT 3 depending regulation of excessive and immoderate proinflammatory cytokine reaction after trauma is more successful in this subcollective by mediating IL-10 induced inhibitory effects. One reason for missing significance might be that it is a crucial point to define cutoffs in order to form dichotomous clinical groups. As there is no information or recommendation for any ISS limit to our knowledge, we chose the median ISS value (41 points) to distinguish between moderate and severe injury.

### 4.3. SOCS 1 mRNA Expression

Considering the dynamic of SOCS 1 mRNA expression patterns 72 h after major trauma in all patients, SOCS 1 levels were significantly downregulated in the posttraumatic course of time. We could also state a significant decrease in three subgroups, as severely and less severely injured patients as well as survivors showed a decrease of SOCS 1 mRNA expression compared to initial values. SOCS 1 is implicated in the suppression of inflammation by regulating innate immune cells and nonimmune cells via interaction with Janus kinases and prevention of catalytic activity [9, 12]. Therefore, we would have expected an upregulation of SOCS proteins depending on high-measured posttraumatic IL-10 and STAT 3 levels. But surprisingly, SOCS 1 mRNA expression decreased after polytrauma in our sample. Several studies have highlighted important SOCS functions as negative feedback proteins limiting chemokine-induced migration of immune cells [21, 22]. Probably, our results might be understood as a failed downregulation of cytokine signalling by a missing mRNA upregulation of SOCS 1. Another hypothetical explanation for our findings might be the fact that there are possible discrepancies regarding mRNA expression levels and protein expression levels. Expected changes in biological active protein levels might not be easily and necessarily reflected in mRNA gene expression levels.

### 4.4. SOCS 3 mRNA Expression

In all patients, SOCS 3 mRNA expressions showed the highest level on admission. Ogle et al. demonstrated that IL-10 and SOCS 3 mRNA expression profiles were elevated after thermal injury in their samples [12]. Our data state a significant overexpression of IL-10 following major trauma and additionally a very high, immediate expression of SOCS 3, followed by a decrease until 24 h after injury. In our study, this posttraumatic decrease of SOCS 3 mRNA levels (6–72 h) was accompanied by a significant increase of STAT 3 mRNA levels directly after trauma (6 h) and a further nonsignificant increase (12–72 h). Normally, JAK-STAT-pathway induces the expression of SOCS 3 in order to turn off IL-6 signalling. Our study's findings demonstrated an anticyclical kinetic of mRNA expression profiles of STAT 3 and SOCS 3. Potentially, our result can be interpreted as an inactivation of this aforementioned feedback loop in a subset of posttraumatic immune disorder. In this context, “SOCS-3 silencing” might permit constitutive STAT 3 signalling.

Remarkably, SOCS 3 mRNA expression showed distinct higher levels in nonsurvivors regarding the whole posttraumatic period with a significant overexpression of SOCS 3 in nonsurvivors six hours after trauma. Following these results, the overproduction of SOCS 3 in nonsurvivors in our study was connected with adverse outcome. In a mouse model SOCS 3 deficiency seemed to protect mice from endotoxemia; evidently, they benefit from the reduction of inflammatory cytokine response due to enhanced anti-inflammatory effect of STAT 3 [23]. In our case, SOCS 3 mRNA overexpression might be a sign for the limited beneficial immune-damping effect of STAT 3 and is thereby associated with adverse outcome.

### 4.5. Patients

In terms of gender, age, injury severity, trauma mechanism and mortality our sample is comparable to other reported groups (DGU TraumaRegister, 2012). As the initial posttraumatic period appears to be predicting for the development of SIRS and subsequent clinical outcome, the reproduction of the course of immunological events within this “small window” requires serial examinations to capture the underlying complexity of posttraumatic immune response [24, 25].

### 4.6. Methods

Separation of granulocytes was performed using anti-CD15 antibody-coated micromagnetic beads (Miltenyi Biotech, Auburn, CA, USA) in accordance with the manufacturer's instructions. This method is known to reproduce a high purity of the isolated cell fraction [26]. Different methods reported in the literature like centrifugation following density gradient have been shown to lead to higher immune cells activation [27–29]. An alternative negative immune magnetic separation technique produces a highly selective cell separation but is unfortunately associated with only small cell recovery [30]. Both the methods for nuclear mRNA and cDNA preparation and qPCR has often been performed and described in the literature and they are known to be well-established and valid methods [31, 32]. In our study, we examined mRNA expression patterns in neutrophil granulocytes, as their key role in innate immune response after severe injury has often been constrained. With 50–60% they cover the main part of circulating leukocytes and influence posttraumatic immune disorder leading to organ dysfunction of trauma patients via cytokine signalling [33].

### 4.7. Limitations of This Study

One limitation to our study is that we primarily focused on mRNA expression profiles in one cell type (PMNs). Regarding our presented results, the evaluated mRNA gene expression levels might have limited expressiveness concerning the biological function, as mRNA levels do not necessarily correlate with protein levels. Particularly cytokines such as IL-10 underlie several posttranscriptional regulation processes, which articulately influence later protein concentration levels. However, advantages in studying gene expression levels and profiles are the detection of early intracellular changes of the transcriptome. To further elucidate the whole complexity of this pathway, protein level analyses and investigations on posttranslational protein modifications will give important further information and are currently underway.

## 5. Conclusion

We present a serial, sequential investigation in human neutrophil granulocytes of major trauma patients evaluating the profiles of mRNA expression of IL-10, STAT 3, SOCS 1, and SOCS 3. Posttraumatically, immune disorder is accompanied by a significant increase of IL-10 and STAT 3 mRNA expression (6 h), whereas SOCS 1 mRNA levels decrease after injury. We could furthermore show that death after trauma is associated with higher SOCS 3 mRNA levels already at 6 h after trauma. To support our results further investigations have to focus on protein levels of STAT/SOCS family in terms of posttraumatic immune imbalance.

## Figures and Tables

**Figure 1:**
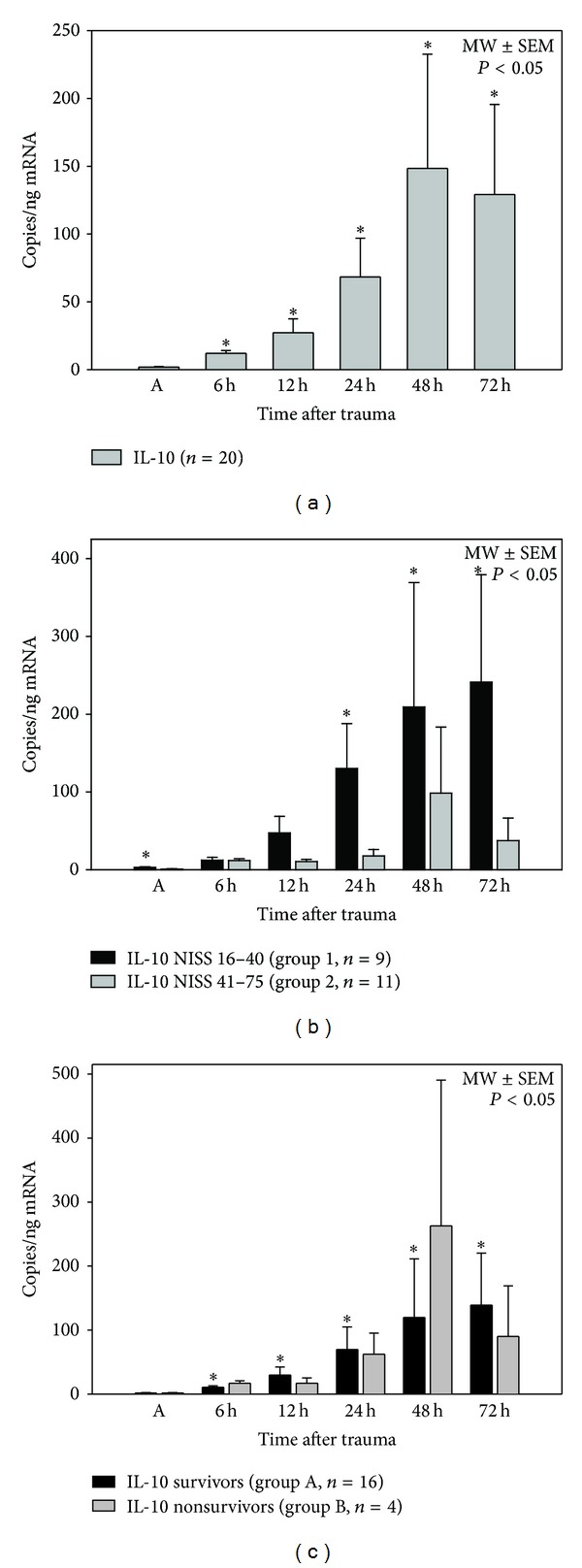
Posttraumatic IL-10 mRNA expression profile after major trauma in all patients (a), depending on injury severity (NISS, (b)) and depending on outcome (survival > 90 days, (c)), *P < 0.05 SNK-test. IL-10 mRNA expression after blunt multiple trauma increased significantly in the early posttraumatic period (6 h–72 h) compared to IL-10 mRNA expression immediately after trauma on admission (A). Patients with a NISS less than 41 points expressed significantly higher IL-10 mRNA levels on admission (A), 24 h, 48 h, and 72 h after trauma, compared to patients with a NISS between 41 and 75 points. Patients who survived the traumatic event showed a significant increase of IL-10 mRNA expression on each time point (6 h–72 h) compared to the IL-10 level detected on admission (A).

**Figure 2:**
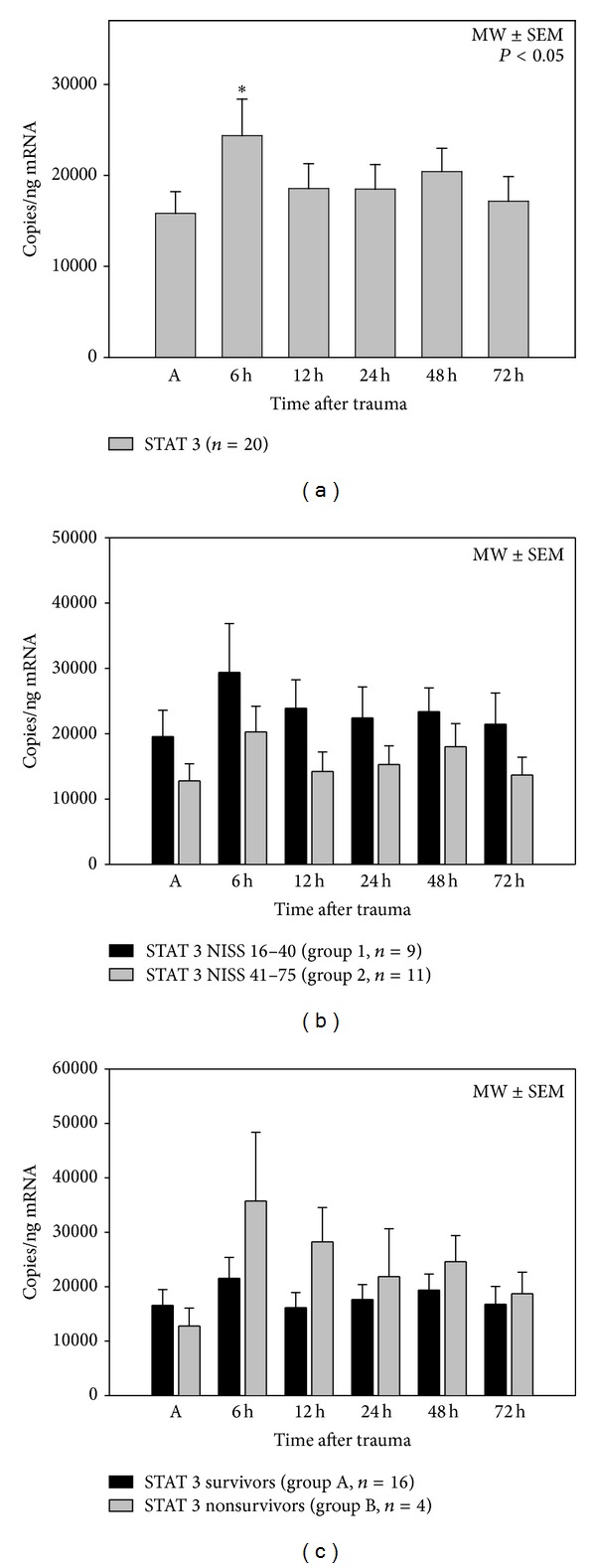
Posttraumatic STAT 3 mRNA expression profiles after major trauma in all patients (a), depending on injury severity (NISS, (b)) and depending on outcome (survival > 90 days, (c)), *P < 0.05 SNK-test. STAT 3 mRNA expressions showed increasing expression levels posttraumatically with a significant maximum level 6 h after the traumatic event in all patients (*n* = 20). STAT 3 mRNA expressions were lower in patients with a NISS between 41 and 75 points compared to patients with a NISS less than 41 points. Nonsurvivors showed a higher STAT 3 expression level within the whole posttraumatic period without significant differences.

**Figure 3:**
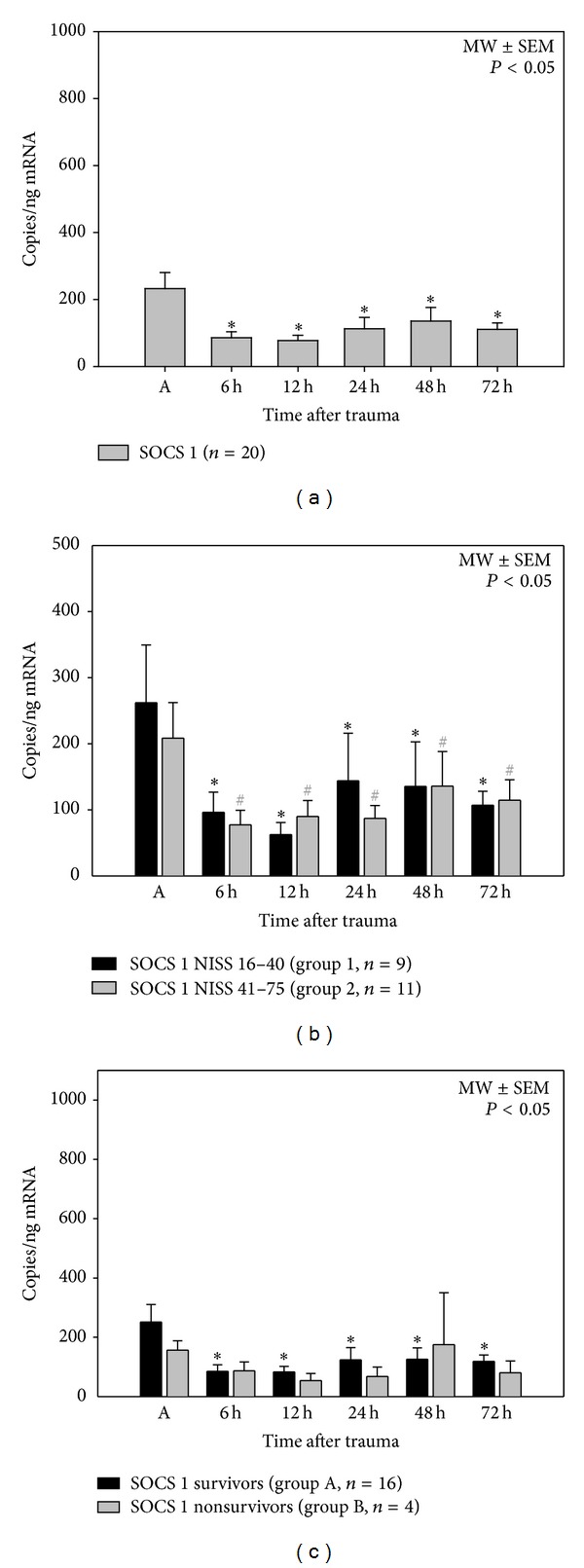
Posttraumatic SOCS 1 mRNA expression profiles after major trauma in all patients (a), depending on injury severity (NISS, (b)) and depending on outcome (survival > 90 days, (c)), *P < 0.05 SNK-test, ^#^ *P* < 0.05  *U* test. SOCS 1 mRNA expression levels significantly decreased after 6 h–72 h compared to SOCS 1 levels measured on admission (A). SOCS 1 mRNA levels of both subgroups (groups 1 and 2) decreased significantly in the early posttraumatic period (6 h–72) compared to SOCS 1 levels on admission (A). There was no significant difference between the subgroups (group 1/group 2). SOCS 1 mRNA levels significantly decreased in survivors (group A) in the posttraumatic observation period (6 h–72 h) in comparison to SOCS 1 mRNA expression detected on admission (A).

**Figure 4:**
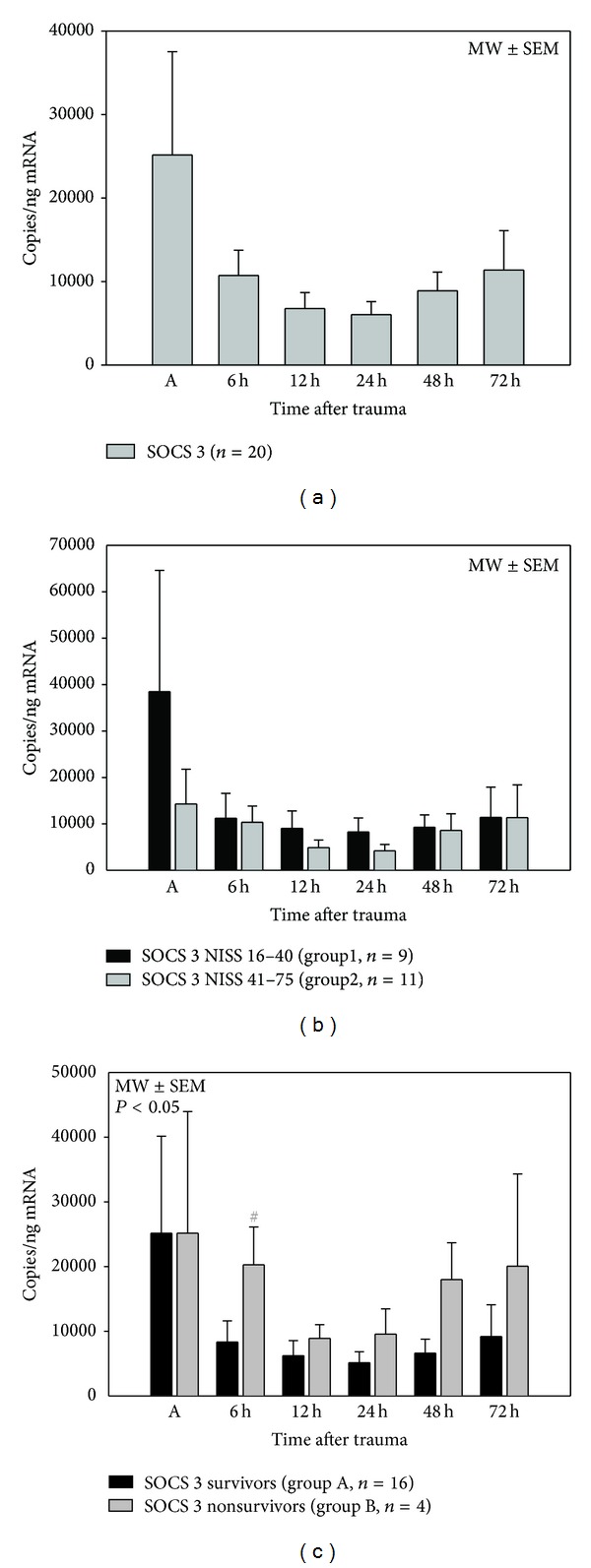
Posttraumatic SOCS 3 mRNA expression profiles after major trauma in all patients (a), depending on injury severity (NISS, (b)) and depending on outcome (survival > 90 days, (c)), ^#^ *P* < 0.05 *U* test. SOCS 3 mRNA expressions showed a high level on admission, followed by a decrease within the first 24 h after trauma in all polytraumatized patients. After the first day, mRNA expression levels increased again until 72 h after injury. Regarding SOCS 3 mRNA expression profiles depending on injury severity, there were no statistically significant differences between groups 1 and 2. Nonsurvivors (group B, *n* = 4) showed a significantly higher SOCS 3 mRNA expression level than survivors 6 h after the traumatic event.

**Table 1:** Clinical data of all patients (*n* = 20).

No.	Age	Gender	NISS	Outcome
1	34	M	57	S
2	39	F	50	S
3	32	F	66	S
4	63	F	27	Ns
5	62	M	27	S
6	45	M	19	S
7	51	M	29	S
8	25	F	57	S
9	57	M	57	S
10	51	F	57	Ns
11	39	M	17	S
12	42	F	41	S
13	40	M	41	S
14	15	M	48	S
15	32	M	43	S
16	49	M	33	Ns
17	32	M	48	Ns
18	28	F	34	S
19	39	M	24	S
20	33	M	34	S

M: male.

F: female.

S: survivors.

Ns: nonsurvivors.
